# Reducing the Salt Added to Takeaway Food: Within-Subjects Comparison of Salt Delivered by Five and 17 Holed Salt Shakers in Controlled Conditions

**DOI:** 10.1371/journal.pone.0163093

**Published:** 2016-09-26

**Authors:** Louis Goffe, Wendy Wrieden, Linda Penn, Frances Hillier-Brown, Amelia A. Lake, Vera Araujo-Soares, Carolyn Summerbell, Martin White, Ashley J. Adamson, Jean Adams

**Affiliations:** 1 Institute of Health & Society, Newcastle University, William Leech Building, Medical School, Newcastle upon Tyne, NE2 4HH, United Kingdom; 2 Fuse–the Centre for Translational Research in Public Health, Newcastle upon Tyne, United Kingdom; 3 Obesity Research Group, School of Medicine, Pharmacy & Health, Wolfson Research Institute, Durham University, Queen’s Campus, Stockton on Tees, TS17 6BH, United Kingdom; 4 Centre for Public Policy & Health, School of Medicine, Pharmacy & Health, Wolfson Research Institute, Durham University, Queen’s Campus, Stockton on Tees, TS17 6BH, United Kingdom; 5 Centre for Diet and Activity Research, MRC Epidemiology Unit, University of Cambridge School of Clinical Medicine, Box 285, Institute of Metabolic Science, Cambridge Biomedical Campus, Cambridge, CB2 0QQ, United Kingdom; University of Kansas Medical Center, UNITED STATES

## Abstract

**Objectives:**

To determine if the amount of salt delivered by standard salt shakers commonly used in English independent takeaways varies between those with five and 17 holes; and to determine if any differences are robust to variations in: the amount of salt in the shaker, the length of time spent shaking, and the person serving.

**Design:**

Four laboratory experiments comparing the amount of salt delivered by shakers. Independent variables considered were: type of shaker used (five or 17 holes), amount of salt in the shaker before shaking commences (shaker full, half full or nearly empty), time spent shaking (3s, 5s or 10s), and individual serving.

**Setting:**

Controlled, laboratory, conditions.

**Participants:**

A quota-based convenience sample of 10 participants (five women) aged 18–59 years.

**Main Outcome Measures:**

Amount of salt delivered by salt shakers.

**Results:**

Across all trials, the 17 holed shaker delivered a mean (SD) of 7.86g (4.54) per trial, whilst the five holed shaker delivered 2.65g (1.22). The five holed shaker delivered a mean of 33.7% of the salt of the 17 holed shaker. There was a significant difference in salt delivered between the five and 17 holed salt shakers when time spent shaking, amount of salt in the shaker and participant were all kept constant (p<0.001). This difference was robust to variations in the starting weight of shakers, time spent shaking and participant shaking (ps</ = 0.001).

**Conclusions:**

Five holed salt shakers have the potential to reduce the salt content of takeaway food, and particularly food from Fish & Chip shops, where these shakers are particularly used. Further research will be required to determine the effects of this intervention on customers’ salt intake with takeaway food and on total dietary salt intake.

## Background

Takeaway food consumption is common in developed countries. Around one-fifth of adults and children in the UK eat takeaway food at home at least once per week.[1] Eating takeaway food at home is more common in children, but not adults, living in more deprived areas.[1] Consumption of takeaway food may be even higher in other countries.[2, 3] Although population data is unavailable, when takeaway food eaten in other locations than home is taken into account, takeaway food is likely to represent a substantial element of the UK diet. One study of UK adolescents living in a deprived urban area found that almost 75% of them consumed any food or drink from fast-food outlets at least once per week.[4] Food prepared out-of-home is, overall, less healthful than food prepared at home[5] and the diets of those who eat more out-of-home food tend to be of poorer nutritional quality.[5, 6]

In England, the takeaway ‘foodscape’ is diverse, but independent outlets tend to be much more common than chain or franchise outlets.[7] Traditional British ‘Fish & Chip Shops’, serving battered and fried white fish with chipped and fried potatoes as their core offering, account for up to one-third of independent takeaways.[8] Aside from other nutrients, food from independent English takeaways is high in salt.[9–11] One study found that the median salt content of one standard portion of fish & chips, before addition of discretionary salt, was 3.0g (IQR: 2.4–4.8)[10]–equivalent to half of the recommended maximum daily intake for adults of 6g.[12] The salt content of other typical dishes served by independent takeaways ranged from 2.2–12.9g.[10] The salt content of fast and takeaway foods in other countries has also been reported to be high.[13–15] Discretionary salt added by servers as they serve and package food, as well as by consumers, would further increase salt content. Reducing salt intake has been associated with reduced blood pressure and incidence of stroke in systematic reviews.[16, 17]

**Fig 1:**
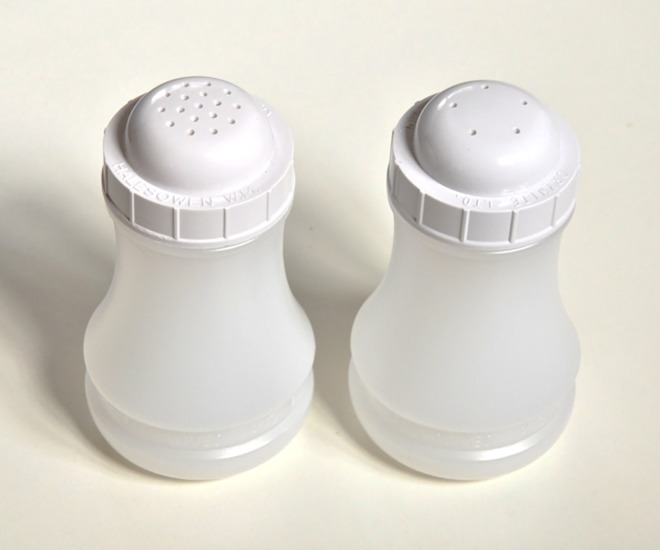
17 (left) and five (right) holed salt shakers used in UK Fish & Chip shops. Image credit: Martin White.

Local government officials in some parts of England are taking action to improve the nutritional quality of food from independent takeaways.[18] One method that aims to reduce the salt content of takeaway food is replacing standard, 17-holed, salt shakers (17HSS) with equivalents with only 5 holes (see **Fig 1**).[19] The five-holed salt shaker (5HSS) attempts to reduce discretionary salt added by servers and–if provided for customer use–consumers. They build on observational findings that discretionary salt use is related more to the size and number of holes in salt shakers, than demographic characteristics.[20]

Five-holed salt shakers were first developed and introduced in Gateshead, in the North East of England, where they were offered, free of charge, to all independent Fish & Chip shops in the area in around 2006. Since then, they have been used in a number of local government initiatives across the country.[19] Whilst 5HSS have been particularly associated with Fish & Chip shops, in some areas their use has been encouraged across the takeaway sector.[18] Although we are not aware of 5HSS being used outside of the UK, they may be useful and appropriate in other settings.

Some evidence suggests that 5HSS tend to be acceptable to takeaway owners.[19] High acceptability is likely to facilitate widespread implementation. Anecdotal, but no formal, evidence suggests that the 5HSS deliver less salt than 17HSS.[19]

We conducted four experiments to determine the salt delivered by 5HSS and 17HSS in controlled conditions; and whether any differences were robust to variations in: the amount of salt in the shaker, the length of time spent shaking, and the person serving.

## Methods

Throughout, the dependent variable was the amount of salt delivered. Independent variables were: type of shaker used (5HSS or 17HSS), amount of salt in the shaker before shaking commences (shaker full, half full or nearly empty), time spent shaking (3s, 5s or 10s), and the individual serving.

### Materials

One 5HSS and one 17HSS produced by Drywite Limited were used and filled with Q Table Salt–supplied by a large regional takeaway supplier and commonly used across the sector. The amount of salt used in each trial was determined by weighing shakers before and after each trial using scales (MyWeigh i2600) accurate to 0.1g.

### Experiment 1: does the amount of salt delivered vary between shakers?

The only independent variable that varied in experiment 1 was type of shaker used (5HSS or 17HSS). Amount of salt in the shaker, and time spent shaking were set to the ‘mid-values’: half full (salt plus shaker weighed 240g) and 5s. One participant was asked to shake each salt shaker for 5s. A count-down timer was used with an audible 3-2-1 lead-in so that the participant knew when to start shaking. An audible tone also indicated when the participant should stop shaking. No further instructions were given for how shaking should be conducted. Salt shakers were refilled between trials. Salt shakers were trialled alternatively. There were ten trials per condition and two conditions: 5HSS and 17HSS. Thus, the 5HSS was shaken, followed by the 17HSS, then the 5HSS, then the 17HSS until ten trials of each shaker had been completed. The participant was not informed of how much salt was delivered on each trial, but they were given limited information on the purpose of the study. Specifically, the information sheet they were provided with stated “We are inviting you to take part in the Salt Shaker research study that is exploring the amount of salt delivered by two different shakers.”

### Experiment 2: is the difference in salt delivered robust to changes in the amount of salt in the shaker?

In this experiment the salt shaker used and the amount of salt in the shaker before shaking commenced varied; time spent shaking was held constant at 5s. The procedure in experiment 1 was repeated twice: firstly using nearly empty salt shakers (salt plus shaker weighed 100g); and secondly using nearly full salt shakers (salt plus shaker weighed 380g). There were ten trials per condition and four conditions: 5HSS nearly empty, 5HSS nearly full, 17HSS nearly empty, and 17HSS nearly full. Nearly empty 5HSS and 17HSS were trialled alternatively and then nearly full 5HSS and 17HSS were trialled alternatively. The same participant who conducted experiment 1 performed all trials.

### Experiment 3: is the difference in salt delivered robust to changes in time spent shaking?

In this experiment the salt shaker used and time spent shaking per trial varied; amount of salt in the shaker before shaking commenced was held constant at half full. The procedure used in experiment 1 was repeated twice: with the participant shaking for 3s and 10s per trial. There were ten trials per condition and four conditions: 5HSS for 3s, 5HSS for 10s, 17HSS for 3s and 17HSS for 10s. The 5HSS was trialled alternatively for 3s and 10s, followed by the 17HSS alternatively for 3s and 10s. The same participant (who conducted experiments 1 and 2) performed all trials and was not informed of how much salt was delivered on each trial.

### Experiment 4: is the difference in salt delivered robust to changes in the person shaking?

In this experiment the salt shaker used and the participant varied; time spent shaking and amount of salt in the shaker before shaking commenced were held constant at 5s and half full. A convenience sample of ten participants, aged 18 years or older was recruited. Quota sampling was used to ensure at least one male and one female participant in each of the following age ranges: 18–29 years, 30–39 years, 40–49 years and 50–59 years. Each participant performed the procedure used in experiment 1.

### Data analysis

Differences in the amount of salt delivered between the two shakers were compared using repeated measures ANOVA tests. One-way tests were used with data from experiments 1–3, and a two-way test with data from experiment 4. All analyses were conducted in Stata SE v13.0 (see **S1 Appendix**).

### Procedure and ethics

Ethical permission was granted by Newcastle University’s ethics committee. Participants were provided with a written information sheet and completed a written informed consent form before any trials began. Participants were not misled in any way. Experiments took place in May-July 2015.

### Data sharing

The full dataset is available from the corresponding author. The statistical code is provided in S1 Appendix. Consent was not obtained for data sharing, but personal identifiable data was not collected, and the risk of identification is low.

## Results

**Table 1** shows the results of all four experiments. Across all trials, the 17HSS delivered a mean (SD) of 7.86g (4.54) per trial, whilst the 5HSS delivered 2.65g (1.22). The 5HSS delivered a mean of 33.7% of the salt of the 17HSS.

**Table 1:** difference in salt delivered by five versus 17 holed salt shakers.

	Start weight (g)	Time shaking (s)	Participants (n)	Trials per participant per shaker (n)	Salt delivered (g), mean (SD)		5HSS as % of 17HSS*	ANOVA F(df), p-value
					5HSS	17HSS		
Exp. 1	240	5	1	10	1.12 (0.32)	2.29 (0.65)	48.9	F(1,9) = 30.79, p < 0.001
Exp. 2	100	5	1	10	1.92 (0.32)	5.81 (0.68)	32.9	F(1,9) = 475.31, p < 0.001
	380	5	1	10	2.13 (0.31)	5.43 (1.12)	39.2	F(1,9) = 132.80, p < 0.001
Exp. 3	240	3	1	10	1.58 (0.39)	3.84 (0.70)	41.1	F(1,9) = 224.89, p < 0.001
	240	5	1	10	2.63 (0.31)	6.75 (1.15)	39.0	F(1,9) = 165.05, p < 0.001
	240	10	1	10	4.45 (0.45)	11.17 (1.20)	39.8	F(1,9) = 313.21, p < 0.001
Exp. 4	240	5	10	10	2.94 (1.29)	9.01 (4.81)	32.6	F(1,156) = 14.93, p = 0.001
All	—	—	10		2.65 (1.22)	7.86 (4.54)	33.7	—

*Note*. 5HSS: five holed salt shaker; 17HSS: 17 holed salt shaker;

*Mean salt delivered by 5HSS as % of mean delivered by 17HSS.

There was a significant difference in salt delivered between the 5HSS and 17HSS in experiment 1 when time spent shaking, amount of salt in the shaker and participant were all kept constant. This difference was robust to variations in the starting weight of shakers, as well as time spent shaking and participant shaking explored in experiments 2–4.

## Discussion

### Summary of results

This is the first documented study we are aware of exploring differences in salt delivered by salt shakers commonly encouraged in independent takeaways in England. We compared the standard 17HSS to the newer 5HSS. Across all experiments, the 5HSS delivered around 34% of the salt delivered by the 17HSS. This difference was robust to changes in the starting fullness of shakers, the length of time spent shaking and the person serving.

### Strengths and limitations of methods

We considered a number of variables that may influence how much salt is delivered by salt shakers: starting fullness of shaker, length of time spent shaking, and person shaking. We focused on length of time spent shaking, rather than number of shakes, as our observations of real-life practice suggest that shaking a salt shaker is a continuous action, rather than a series of discrete actions. Our anecdotal observations in Fish & Chip Shops also suggest that median time spent shaking is around 4-5s, ranging from around 1-10s, indicating that the range of times we chose are largely reflective of practice. We conducted 10 trials of each condition, and recruited a range of different individuals for experiment 4 to increase the reliability of our results.

Participants were only semi-blinded to the purpose of the experiment. They were aware that we were investigating how much salt different shakers delivered. But they were not aware which was the ‘new’ shaker or which was proposed to deliver less salt. Given that participants could also see how much salt was being delivered (although they were not informed of how much salt was actually delivered), this may have had some influence on their shaking behaviour.

Experiments 1–3 were all conducted by the same individual and in series. It is possible that this subject was more careful in their shaking, and less tired, during experiment 1 than in later experiments. However, there remained clear differences between salt shakers in all experiments, suggesting this did not impact substantially on the results.

Salt shakers were trialed alternatively in all experiments–that is the 5HSS was trialed, then the 17HSS, then the 5HSS, then the 17HSS until 10 repeats of each had been conducted. If subjects tired during testing, this may have differentially effected the different shakers. However, by alternating shakers throughout, this is likely to have a small effect, if any.

We were not able to take account of all variables that may influence how much salt is delivered in practice. These include: customer preference, humidity leading to potential clogging of shakers, and any shop-specific special procedures. Our results represent controlled conditions and may not be generalizable to salt shaker use in practice.

The sample size in all experiments may appear ‘low’. The main risk of a small sample size is of type 2 error–that is, failing to identify a difference where one exists. As we identified a difference in all comparisons, there is no risk of type 2 error. However, it is possible that our results are subject to type 1 error–that is, identifying a difference where one does not exist. The main method for reducing type 1 error is to reduce the threshold p-value taken to indicate statistical significance. All of our p-values were ≤0.001 –indicating that type 1 error will occur in 0.1%, or fewer, tests. Given we have conducted 7 tests, the overall chance of type 1 error is less than 0.7%. As such, our results are very unlikely to be subject to type 1 error.

### Interpretation of results and implications for policy, practice and research

Our results are encouraging for the increasing number of English local government areas and independent takeaways who promote, or use, the 5HSS to reduce the salt content of takeaway food. They may also be a useful prompt for those working to reduce the salt content of takeaway food in other countries to consider how 5HSS could work in other settings. Although our intention was not to determine under what conditions the least amount of salt is delivered, our results do suggest that less salt is delivered when shakers are half full, compared to nearly empty or nearly full. It is not clear how practical this finding could be in practice. Unsurprisingly, shaking for less time also resulted in less salt delivered.

We cannot conclude from our results that the 5HSS will necessarily be associated with less salt consumed with takeaway food. For example, in real-life settings, servers may shake for longer with the 5HSS,[21] or customers may ask for, or add their own, additional salt. There is some anecdotal evidence to suggest these, unintended, consequences do occur.[18] Further research is required to confirm that the 5HSS is associated with less salt added to takeaway food, less salt consumed with takeaway food, and to explore any impact on customers’ total diets.

The results of experiment 4 showed substantial between-person variation in the amount of salt delivered by the 5HSS and 17HSS. Indeed, between-subjects variance was 1.57 for the 5HSS and 23.04 for the 17HSS, whilst within-subjects variance was 0.26 for the 5HSS and 1.87 for the 17HSS. Whilst, overall, the 5HSS delivered less salt than the 17HSS in experiment 4, the salt delivered by some individuals using the 5HSS was more than that delivered by others using the 17HSS. Between-person variation should, therefore, also be expected in practice. Substantial variation in salt content of takeaway food has been previously documented[10] and this may reflect both variations in recipes and serving practice. The variance figures reported above give variance ratios (between-subjects variance/within-subjects variance) of 6.04 for the 5HSS and 12.32 for the 17HSS–indicating proportionally greater between-subjects than within-subjects variance for the 17HSS than the 5HSS. The 5HSS may help standardise, as well as reduce, the amount of salt added to food.

The 5HSS only addresses discretionary salt added by servers, and possibly customers, to takeaway food. The 5HSS does not address the relatively high levels of salt added to these foods in preparation.[10, 12] Further interventions may be required to help takeaways reformulate recipes to reduce salt added during preparation. Reformulation to reduce salt content has been successful in the wider UK food industry.[22] Other, wider, initiatives are also be required to tackle salt consumption holistically.

## Conclusion

Five holed salt shakers delivered around 34% of the salt of 17HSS in controlled conditions. This difference was robust to variations in: the amount of salt in the shaker, the length of time spent shaking, and the person serving. This confirms the potential of the 5HSS as a method to reduce the salt content of takeaway food, and particularly food from Fish & Chip shops, where these shakers are particularly used. Further research will be required to determine the effects of this intervention on customers’ salt intake from takeaway food and total dietary salt intake.

## Supporting Information

S1 Appendix Statistical code. (DOCX)
