# Supplementary material for: Reducing the Salt Added to Takeaway Food: Within-Subjects Comparison of Salt Delivered by Five and 17 Holed Salt Shakers in Controlled Conditions
Source: PLoS One. 2016 Sep 26;11(9):e0163093. doi: 10.1371/journal.pone.0163093 (PMC5036815; doi:10.1371/journal.pone.0163093)
Supplement: S1 Appendix — (DOCX) [file pone.0163093.s001.docx]

**S1 Appendix. Statistical code**

***exp 1

use "U:\My Documents\Jean\Current projects\SPHR Foodscape\salt shaker exp 1.dta", clear

gen repeatid2=repeatid

recode repeatid2 (1=1) (3=2) (5=3) (7=4) (9=5) (11=6) (13=7) (15=8) (17=9) (19=10) (2=1) (4=2) (6=3) (8=4) (10=5) (12=6) (14=7) (16=8) (18=9) (20=10)

anova saltused shaker repeatid2, repeated(repeatid2)

bysort shaker: sum saltused

***exp 2

use "U:\My Documents\Jean\Current projects\SPHR Foodscape\salt shaker exp 2.dta", clear

gen repeatid2=repeatid

recode repeatid2 (1=1) (3=2) (5=3) (7=4) (9=5) (11=6) (13=7) (15=8) (17=9) (19=10) (2=1) (4=2) (6=3) (8=4) (10=5) (12=6) (14=7) (16=8) (18=9) (20=10) (21=1) (22=1) (23=2) (24=2) (25=3) (26=3) (27=4) (28=4) (29=5) (30=5) (31=6) (32=6) (33=7) (34=7) (35=8) (36=8) (37=9) (38=9) (39=10) (40=10)

bysort startweight: anova saltusefive shaker repeatid2, repeated(repeatid2)

bysort startweight shaker: sum saltusefive

***exp 3

use "U:\My Documents\Jean\Current projects\SPHR Foodscape\salt shaker exp 3.dta", clear

gen repeatid2=repeatid

recode repeatid2 (1=1) (3=2) (5=3) (7=4) (9=5) (11=6) (13=7) (15=8) (17=9) (19=10) (2=1) (4=2) (6=3) (8=4) (10=5) (12=6) (14=7) (16=8) (18=9) (20=10)

anova saltuse3 shaker repeatid2, repeated(repeatid2)

anova saltuse5 shaker repeatid2, repeated(repeatid2)

anova saltuse10 shaker repeatid2, repeated(repeatid2)

bysort shaker: sum saltuse3 saltuse5 saltuse10

***exp 4

use "U:\My Documents\Jean\Current projects\SPHR Foodscape\salt shaker exp 4.dta", clear

gen repeatid2=repeatid

recode repeatid2 (1=1) (3=2) (5=3) (7=4) (9=5) (11=6) (13=7) (15=8) (17=9) (19=10) (2=1) (4=2) (6=3) (8=4) (10=5) (12=6) (14=7) (16=8) (18=9) (20=10)

anova saltuse5 shaker/personid|shaker repeatid2 shaker#repeatid2, repeated(repeatid2)

bysort shaker: sum saltuse5
